# Hypertension and Ovarian Cancer: A Case-Control Study in Saudi Arabia

**DOI:** 10.7759/cureus.35294

**Published:** 2023-02-22

**Authors:** Bandar M Alrobaiq, Rashed S Alharbi, Faisal S Alhoshan, Mohammed A Alnasyan, Abdulrahman Alahideb, Aamir Omair

**Affiliations:** 1 College of Medicine, King Saud Bin Abdulaziz University for Health Sciences College of Medicine, Riyadh, SAU; 2 Medical Education, King Saud Bin Abdulaziz University for Health Sciences, Riyadh, SAU

**Keywords:** high-density lipoprotein (hdl), body mass index (bmi), triglyceride, ovarian cancer, hypertension

## Abstract

Background

There is limited evidence that evaluates the association between hypertension and ovarian cancer. The study aims to investigate the association between ovarian cancer and hypertension, the difference in lipid profile, and the association between body mass index (BMI) and ovarian cancer.

Methods

We conducted a case-control study at King Abdelaziz Medical City (KAMC), oncology department. All Saudi female patients who were diagnosed with primary ovarian cancer admitted to the oncology department at KAMC from 2016 to 2019 were selected. The data were collected from medical records of patients of the KAMC by chart review using The Ministry of National Guard Health Affairs BESTCare database.

Results

A total of 137 Saudi female patients diagnosed with ovarian cancer attending to gynecology and oncology center in KAMC from 2016 to 2019 were included in this study. The mean age of participants was 57 in cases and 56 in controls with a mean BMI of 29.64 in cases and 31 in controls. There were 63 obese cases, therefore, the proportion of obesity was 46%. Approximately one-third of cases were overweight (28%) while one-fourth (26%) of them were underweight or normal weight. Roughly two-thirds of cases were hypertensive with an overall proportion of 66 % (95% confidence interval (CI) 58-74) while one-third of controls were hypertensive with an overall proportion of 32%. Cases were having significantly higher triglycerides (p=0.03) and lower high-density lipoprotein (HDL) (p=0.001) than controls. The significant variables were analyzed using logistic regression. It was found that hypertensive subjects were 10.06 times more likely (95% CI: 4.88-20.71) to be associated with the cases as compared to controls (p<0.001). Also, an increase in BMI was significantly associated with being a case with OR = 1.07 (95% CI: 1.02-1.12; p=0.004).

Conclusion

In conclusion, hypertension, elevated BMI, higher triglycerides, and lower HDL were significantly associated with ovarian cancer.

## Introduction

Ovarian cancer occurs when cells grow abnormally forming tumors in the ovary. Ovarian cancer has been estimated to be the seventh most common cancer diagnosed among women in the world and the fifth most common cancer-related death among women in the United States. Although ovarian cancer is less prevalent than breast cancer, it is three times more lethal [1,2]. The prevalence of ovarian cancer globally was estimated to be 3.6% of cancer cases and 4.3% of cancer-related deaths in female patients [3]. The number of identified cases of ovarian cancer among Saudi female patients was estimated to be 991 from 2001 to 2008, and Riyadh was the highest incidence rate of ovarian cancer, while the lowest incidence rate of ovarian cancer was in the northern region of Saudi Arabia [4].

Unfortunately, the incidence rate of ovarian cancer around the world is continuously increasing due to an increase in risk factors, in Saudi Arabia for example, the incidence rate increased by up to 5% from 2001 to 2008 [4,5]. There are potential risk factors for developing ovarian cancer. Family history of ovarian cancer, age, infertility therapy, and hormonal replacement after menopause are possible factors that favor developing ovarian cancer [5,6]. Metabolic syndrome increases the risk of developing cancer types and cancer-related morbidity and mortality [7]. Metabolic syndrome is a collection of risk factors encompassing high blood pressure, elevated blood glucose, raised triglyceride (TG), low high-density lipoprotein (HDL), and abdominal obesity. Obesity is associated with an increased risk of ovarian cancer, particularly in women who are extremely obese and pre-menopausal, however, there was no significant association noticed in post-menopausal women based on a meta-analysis study [8]. On the other hand, a recent meta-analysis study declares that there was an 80% increased risk of ovarian cancer in obese patients in post-menopausal women compared to normal weight, particularly those who do not use hormonal replacement therapy [9]. Multiple studies have investigated the association between lipid profile and ovarian cancer but ended up with different conclusions. For example, a systematic review and meta-analysis study included 12 studies and found that high total cholesterol is significantly associated with ovarian cancer [10]. Contrariwise, total cholesterol and HDL were significantly lower among ovarian cancer patients in multiple studies, based on a meta-analysis study published in 2020 [11]. Finally, there is a moderate increase in the risk of ovarian cancer in diabetic patients based on a meta-analysis study [12].

To our knowledge, there are limited studies that investigate the association between hypertension and ovarian cancer as searched using PubMed and using the following terms (hypertension and ovarian cancer) and/or (metabolic syndrome and ovarian cancer), which necessitates further studies, especially in different populations. The authors aimed to investigate the association between ovarian cancer and hypertension, the difference in lipid profile, and the association between body mass index (BMI) and ovarian cancer.

## Materials and methods

We conducted a case-control study at the oncology department of King Abdelaziz Medical City (KAMC) in 2019. It is a dynamic and progressive entity in comprehensive cancer care. It currently has five medical divisions: Adult Hematology, Adult Medical Oncology, Gynecology Oncology, Pediatric Hematology/Oncology, and Palliative Care. The data were collected from medical records of patients of the KAMC by chart review using The Ministry of National Guard Health Affairs BESTCare database. All Saudi female patients who were diagnosed with primary ovarian cancer admitted to the oncology department at KAMC from 2016 to 2019 were selected. We chose this period because the gynecology oncology center was established in 2016. To assess the association between ovarian cancer and hypertension as well as the difference in lipids and the association of obesity between cases and controls, we selected controls who attended KAMC for the same period of cases with matching age (+/- 5 years) without ovarian cancer and diabetes using the consecutive technique. The independent variables were age and ovarian cancer, whereas the dependent variables were hypertension, overweight, and obesity. Being overweight was defined as a BMI of 25-29.9 kg/m^2^, whereas obesity was defined as a BMI of 30 kg/m^2^ or higher. Both overweight and obesity were identified based on the World Health Organization Classification. Hypertension was defined as a systolic blood pressure of 130 or above and/or diastolic blood pressure of 80 and above based on the American Health Association Classification. Data were analyzed using SPSS (statistical package for social sciences) analysis software version 22 (IBM Corp, Armonk, New York, USA). Continuous variables were described using means and standard deviations, whereas categorical variables were presented as numbers and percentages. we used a T-test to assess if there is a significant difference between the means of the two groups. We used logistic regression analysis to assess the association of hypertension and BMI with ovarian cancer. The study was approved by the institution review board of King Abdullah International Medical Research Center (KAIMRC) (Approval number: SP20/362/R).

## Results

A total of 137 Saudi female patients diagnosed with ovarian cancer attending to gynecology and oncology center in KAMC from 2016 to 2019 were included in this study. The mean age of participants was 57 in cases and 56 in controls with a mean BMI of 29.64 in cases and 31 in controls. There were 63 obese cases, therefore, the proportion of obesity was 46% (Table 1). Approximately one-third of cases were overweight (28%) while one-fourth (26%) of them were underweight or normal. Roughly two-thirds of cases were hypertensive with an overall proportion of 66% while one-third of controls were hypertensive with an overall proportion of 32% (Table 2). The means of TG in cases and controls were 1.34 mmol/L and 1.16 mmol/L, respectively, while the mean of HDL in cases and controls was 1.13 mmol/L and 1.29 mmol/L, respectively (Table 3). The mean of cholesterol and low-density lipoprotein (LDL) were similar as described in Table 3.

**Table 1:** Characteristics of participants

		Group				
		Case (n=136)		Control (n=137)		
		n	%	n	%	p-value
Age group	Mean + SD	57.2 + 16.3		56.9 + 16.4		0.87
BMI	Underweight/Normal weight	35	26%	22	16%	0.14
	Overweight	38	28%	42	31%	
	Obese	63	46%	73	53%	
	Mean + SD	29.64 + 6.65		31.33 + 7.09		0.04

**Table 2:** Proportion of hypertension HTN: hypertension

			Groups		Total
			Case	Control	
HTN	Normotensive	Count	45	93	138
		% within group	33.8%	67.9%	51.1%
	Hypertension	Count	88	44	132
		% within group	66.2%	32.1%	48.9%
Total		Count	133	137	270
		% within group	100.0%	100.0%	100.0%

**Table 3:** Lipid profile of participants and T test

Group		N	Mean	Std. Deviation	p-value
Cholesterol level (mmol/L)	Case	73	4.81	1.03	0.60
	Control	137	4.74	0.96	
Triglycerides (mmol/L)	Case	70	1.34	0.56	0.03
	Control	136	1.16	0.51	
HDL (mmol/L)	Case	75	1.13	0.31	0.001
	Control	137	1.29	0.31	
LDL (mmol/L)	Case	75	3.10	0.97	0.44
	Control	137	3.00	0.89	

Comparing the mean TG levels between cases and controls showed that cases had significantly higher TG levels than controls (p=0.03). Furthermore, cases were having significantly lower HDL than controls (p=0.001) (Table 3).

The significant variables were analyzed using logistic regression. It was found that hypertensive subjects were 10.06 times more likely (95% CI: 4.88-20.71) to be associated with the cases as compared to controls (p<0.001). Also, an increase in BMI was significantly associated with being a case with OR = 1.07 (95% CI: 1.02-1.12; p=0.004) (Table 4).

 

**Table 4:** Logistic regression

	OR	95% CI for OR		p-value
		Lower	Upper	
Hypertensive	10.06	4.88	20.71	<0.001
BMI	1.07	1.02	1.12	0.004

## Discussion

This study investigates the association between ovarian cancer and hypertension, the difference in lipid profile, and the association between BMI and ovarian cancer. The data obtained from this study showed that obesity was significantly associated with ovarian cancer (OR = 1.07 95% CI: 1.02-1.12; p=0.004). This finding was in agreement with the previous studies [8,9]. Although the exact etiology of obesity’s increased risk of developing ovarian cancer is unknown, it is hypothesized that excess body mass increases ovarian cancer partly through the estrogenic effect by increasing the synthesis of estrogen levels in adipocytes. In addition, cases had significantly higher TG levels (p=0.03). Although a significantly higher TG level in ovarian cancer compared to controls was observed in other studies, when age-stratified is applied TG level was insignificant [11]. furthermore, the authors observed that HDL was significantly lower than controls (p=0.001) which is consistent with previous studies [11]. It could be partly explained that since cancer cells have a strong affinity for sterols and lipids, lipid metabolism is a crucial component of cancer signaling [13,14].

Finally, in the present study, hypertension was significantly associated with ovarian cancer (OR: 10 95% CI: 4.88-20.71). Similarly, a case-control study conducted at Tianjin Medical University, China enrolled 573 epithelial ovarian cancer patients and 1146 matched controls and revealed that hypertension was significantly associated with ovarian cancer (OR = 2.423; 95% CI: 1.963-1.2.990) [15]. In addition, a prospective cohort study that enrolled 287320 women from Austria, Norway, and Sweden found that during the follow-up, 644 epithelial ovarian cancer and 388 death from ovarian cancer, and they concluded that there is no association between metabolic syndrome and epithelial ovarian cancer, however, increasing in blood pressure and blood cholesterol increase the risk of mucinous and endometrioid tumors, respectively [16]. On the other hand, a network of case-control study which conducted in Italy enrolled 970 ovarian cancer and 3045 controls suggesting no association between ovarian cancer and hypertension [17]. Hypertension is associated with certain types of cancer. For example, a systematic review and meta-analysis study investigated the association between breast cancer and hypertension and suggested that hypertension is significantly associated with an increased risk of breast cancer (RR: 1.15; 95% CI: 1.08-1.22) [18].

Moreover, in a large prospective pooled cohort study, both treated and untreated, hypertension was associated with an increased likelihood of developing cancer compared with normotensive individuals [19]. Additionally, based on a prospective cohort study by Stocks et al. of roughly 577,800 adults followed for 12 years, there was an association between hypertension and cancer incidence in men and between hypertension and higher cancer mortality in both men and women [19]. The exact mechanism by which hypertension causes cancer is unknown. Animal models suggest dysregulation of apoptosis due to elevated blood pressures contributing to cancer [20,21]. Another possible explanation for overburdened hypertension among ovarian cancer patients is that using certain classes of chemotherapeutic agents is associated with increased blood pressure [22]. For example, bevacizumab (anti-vascular endothelial growth factor) can induce endothelial dysfunction along with decreased nitric oxide bioavailability causing an increase in the vascular tone which contributes to the elevation of blood pressure [21].

This study has methodological limitations as in other studies. Unfortunately, there was no documented information about medications such as chemotherapy and hormonal replacement therapy which can be a confounder. Because of that, it may result in reporting bias. Additionally, diabetes could not be investigated in our study since most of the cases did not have documented blood glucose which also makes it impossible to evaluate the association between metabolic syndrome and ovarian cancer.

## Conclusions

In conclusion, hypertension was significantly associated with ovarian cancer. In addition, low HDL, high TG, and elevated BMI were significantly associated with ovarian cancer.
